# Trichorionic Quadruplet Delivered Beyond 36 Weeks of Gestation: A Case Report and Literature Review

**DOI:** 10.1155/2011/181034

**Published:** 2011-10-20

**Authors:** Fawaz E. Edris

**Affiliations:** ^1^Department of Obstetrics and Gynaecology, University of Um-AlQura, Makkah 21955, Saudi Arabia; ^2^Department of Obstetrics and Gynaecology, International Medical Centre, P.O. Box 2172, Jeddah 21451, Saudi Arabia

## Abstract

Following one year of secondary fertility, a couple conceived with a quadruplet after transfer of three embryos through IVF-ICSI. At 36 weeks and 2 days of gestation, the mother developed gestational induced hypertension and delivered the next day by caesarean section. Pathology confirmed the zygosity to be trichorionic quadramniotic and all four babies were discharged home with their mother on postoperative day 3. Herein, we describe a successfully managed high-risk pregnancy case. A review of the literature was conducted and to our knowledge no other cases with similar criteria ever reached such advanced gestational age.

## 1. Introduction

Quadruplet pregnancy is considered a very high-risk pregnancy due to the multiple potential complications that include, but not limited to, preterm delivery with a median gestational age at delivery ranging between 28 and 31 weeks [1, 2], increased perinatal, neonatal, and infant mortality [2–5], and intrauterine growth restriction [3]. Twin-Twin Transfusion Syndrome (TTTS) is a potential lethal complication that can affect 10% to 30% of Monochorionic twins [6, 7]. Here we report a case of trichorionic quadramniotic quadruplet that was delivered at 36 weeks and 3 days of gestation. To our knowledge no other cases with similar criteria ever reached that advanced gestational age.

## 2. Case Report

A 27 year-old, para 3 lady and her husband presented to our clinic with a one year history of secondary infertility that was labelled as unexplained after confirming normal gonadotrophins levels, hysterosalpingogram, and advanced semen analysis. The couple declined diagnostic laparoscopy and chose to undergo treatment with intrauterine insemination (IUI) after they were counselled about their options of assisted reproductive techniques including IUI and IVF with and without ICSI, and their potential complications including the risk of multiple pregnancy and their potential consequences.

Ovarian stimulation was undertaken with gonadotrophin using a recombinant FSH product (Puregon; Organon, Oss, The Netherlands). The patient overresponded and had developed 13 follicles over the size of 15 mm on stimulation day 13. The couple was counselled to cancel the treatment cycle or switch to IVF provided an LH surge did not occur. The couple chose the latter and ovarian stimulation continued with a higher dose of the same recombinant FSH product after starting a GnRH antagonist (Cetrotide; Serono, Baxter Oncology, Germany). 

When most follicles were in the range of 17 to 22 mm, final oocyte maturation was completed with hCG (Choriomon; IPSA, Lugano, Switzerland). Thirty-six hours later, a vaginal ultrasound-guided oocyte collection was undertaken and a total of 23 oocytes were retrieved. After stripping of the surrounding cumulus oophorus from the oocytes in preparation for ICSI, the embryologist evaluated only 19 oocytes as being mature and each was inseminated by a single sperm. After 18 to 20 hours, 17 oocytes showed pronucleate development and were further evaluated on day 2 and day 3 after oocyte recovery. The remaining 2 unfertilized eggs were discarded.

On day 3, of the 17 embryos, 10 had developed to the 8-cell stage, one to the 7-cell stage, and 6 remained at 2-to-4-cell stage. After extensive counselling, the couple chose to have 3 embryos transferred back to the uterus, understanding the risk of multiple pregnancy and its potential consequences. Three very good quality 8-cell embryos (with grade 3.5 to 4 out of 4) were considered most appropriate for embryo transfer and this was undertaken later that day at around 72 hours after oocyte recovery. Embryo transfer was undertaken without difficulty using Sydney IVF embryo transfer catheter (Catalogue number K-JETS-6019-SIVF, Cook, Limerick, Ireland). The remaining 8 good quality embryos (seven 8-cell and one 7-cell embryos with grade 3 to 3.5 out of 4) underwent cryopreservation using vitrification technique.

On luteal day 18, the patient had a positive pregnancy test with a BhCG level of 747 IU/mL. An ultrasound was performed on luteal day 49, and four intrauterine sacs with embryos each measuring 7 to 10 mm were seen (appropriate for the equivalent gestational age). A normal heartbeat was noted in all embryos. Not surprisingly, the couple was shocked by this news but further discussion and counselling was carried out that day and repeated a week later outlining the possible causes of what have happened, the risks of high order multiple pregnancy, the possible treatment options open to them at that time including selective reduction, the supervision and management of the pregnancy should they choose to continue and the likely options and the gestational age at which delivery might occur. 

At 11 weeks of gestation, the couple was seen and underwent an ultrasound study that confirmed the viability of all four foetuses. Chorionicity was examined by looking at the intersac membranes and they appeared to be trichorionic quadramniotic pregnancy as the dividing membrane between two of the foetuses showed a “T” sign as opposed to a “lambda” sign that was seen in the other dividing membranes between the foetuses. This was expected as it was the most likely explanation for the transformation of those 3 embryos to 4 foetuses. The couple was counselled again about all potential complications of such kind of pregnancy and they were also counselled about the pros and cons and controversies of cervical cerclage and nuchal translucency measurement in high-order multiple pregnancies. They chose not to undergo nuchal translucency measurement as they made up their mind that they will continue this pregnancy no matter what the outcome is. The couple chose to have a cervical cerclage only if all four foetuses remained alive in 2 weeks. At 13 weeks of gestation, and after confirming viability of the 4 foetuses, McDonald cervical cerclage was carried out with no complications. 

The patient was seen every 2 weeks starting at 15 weeks of gestation to rule out the early development of TTTS. A detailed morphology exam (level II ultrasound) showed normal 4 foetuses at 19 weeks of gestation with no major anomalies seen in any of them. At 26 of gestation, the patient had some uterine irritability and two doses of 12 mg betamethasone were given a day a part. Three weeks later, gestational diabetes was ruled out and biophysical profile (BPP) as well as end-diastolic-flow umbilical artery Doppler (EDF-UAD) were started at around 29 weeks of gestation and repeated every 2 weeks. 

At around 33 weeks of gestation, one of the monochorionic foetuses was relatively smaller than the others, with parameters consistent with intrauterine growth restriction (IUGR); however it remained to have normal and concordant amniotic fluid and EDF-UAD. Foetal assessment started to be done on weekly bases in the form of amniotic fluid assessment and EDF-UAD. At 35 weeks and 2 days of gestation, the small foetus remaind to be IUGR, however all four foetuses showed normal interval growth, amniotic fluid, and EDF-UAD. A week later, the patient was found to have a mildly elevated blood pressure (around 142/90 mmHg), but no proteinuria. She was asymptomatic and her investigations including those for HELLP syndrome were normal. Amniotic fluid assessment as well as EDF-UAD of all foetuses continued to be normal with exception to the small foetus that started to have intermittent absent end diastolic flow. 

Given the excellent gestational age reached and the development of those multiple mild complications, delivery was advised and was undertaken by semielective caesarean section the following day at a gestational age of 36 weeks and 3 days. At this, she delivered four healthy infants, three girls and one boy. Their weights were 1785, 1890 grams (gm), for the monochorionic twin, and 2035 and 2225 gm for the other girl and boy, respectively. The APGAR scores at 1 and 5 minutes were 8 and 9, respectively, for all four infants. All four infants were admitted to the Neonatal Intensive Care unit (NICU) for monitoring only. Four hours later, 2 infants were discharged to the normal nursery including the smallest one. The other two were on nasal oxygen cannula (blended oxygen) as they had Transient Tachypnea of Newborn (TTN), but both were discharged to the normal nursery in less than 24 hours. Pathological examination of the placentae confirmed the zygosity of trichorionic quadramniotic quadruplet. All four babies were discharged home with their mother on post-operative day 3. The babies were seen at 2 and 6 months of age for their scheduled immunization shot. All babies were well and with developmental assessment corresponding to their age. Permission was sought and obtained from the couple to publish this report.

## 3. Discussion

Pregnancy with multiples is considered a high-risk pregnancy, with more complications observed as the number of foetuses increase. Compared with mothers of twins, mothers of triplets and quadruplets were more likely to be diagnosed with preterm premature rupture of membranes, have excessive bleeding, delivered at less than 29 weeks of gestation, and to have one or more infants die [8]. The gestational age at delivery for quadruplets varied in different reports, ranging from 28 weeks to 31 weeks [1, 2]. Few reports claimed 34 weeks as the gestational age at which delivery took place [9, 10]. Compared to the above, our case was peculiar in that none of the mentioned complications occurred, and the delivery was not conducted until 36 weeks and 3 days of gestation. 

A review of multiple pregnancies that included 133 foetuses found that there were three stillbirths, seven neonatal deaths, six early neonatal deaths, and one late neonatal death [4]. A larger review that included 1448 quadruplets also confirmed a significantly neonatal, perinatal, and infant mortality rates [11]. The early mortality (combined perinatal and infant mortality) increased significantly with each additional foetus in a dose-dependent fashion, corresponding to relative risks of 2.4 for triplets, 3.3 for quadruplets, and 10.3 for quintuplets in a study that was composed of around 318000 sets of twins [5]. Others have reported similar trends in outcome with a perinatal mortality of 14 for triplets and 36 for quadruplets [12]. In a retrospective study that included 24 sets of quadruplet pregnancies, the quadruplets with at least one monochorionic pair had 5 times higher perinatal mortality than quadrochorionic quadruplet pregnancies [2]. Our case was a quadruplet with one set of monochorionic twin. All four babies were alive and well at birth and remained so till 6 months of age.

Early neonatal and perinatal mortalities were significantly higher in quadruplets and quintuplets than in triplets; however survival of growth restricted foetuses in that study was better than survival of their eutrophic counterparts [13]. Although our babies had 100% survival and none was kept in NICU for more than a day, our IUGR baby was discharged only 4 hours after admission to the NICU, while 2 bigger ones stayed overnight for almost 24 hours, which is in agreement with this study. 

A review that involved 116785 infants including 3448 twins, 99 triplets, and 16 quadruplets, showed a significantly higher rates of major anomalies among triplets and quadruplets [3]. Others have confirmed the same finding [4]. Additionally, some authors showed a significantly higher occurrence of emergent peripartum hysterectomy in multiple gestations compared to singletons [14]. None of our babies had a major anomaly and all were discharged home with their mother 3 days after uncomplicated caesarean section.

Other complications such as gestational diabetes mellitus, abruptio placenta, and premature rupture of membrane were significantly higher in women with triplet pregnancies and quadruplet and higher-order multiple gestations than in women with twin pregnancies in a review that included over 158000 multiple pregnancies [15]. Other smaller reviews observed the same finding [4, 8]. Our case did not sustain any of those complications. 

Retrospective analysis of neonatal data obtained from all Swiss hospitals showed that the median birth weight was 1665 gm for triplets and 1076 gm for quadruplets. The review also showed that respiratory distress syndrome (RDS) was the major morbidity as it was diagnosed in 52% of triplets and 81% of quadruplets [1]. Others have reported similar trends of outcome with RDS occurring in up to 23% of triplets and 65% of quadruplets [12]. The median birth weight for our babies was 1962 gm and none developed RDS.

Our case was a 27-year old with a favourable outcome, although some authors noticed a significantly increased neonatal, perinatal, and infant mortality rates among younger mothers (less than 35-year old) with quadruplets and quintuplets pregnancies compared to those over 35 years [11]. On the other hand, our patient was a parous woman, and that was a good prognostic factor, as some authors found that gestational age at delivery is significantly increased in parous women carrying a multifetal gestation after controlling for other factors that affect gestational age at birth [16].

TTTS is a devastating complication that can affect 10–30% of monochorionic twins [6, 7] and is associated with up to 80% mortality rate in untreated cases [17]. TTTS has been reported as early as 11 weeks in a trichorionic quadruplet pregnancy which was conceived after IVF-ICSI [18]. Our case was lucky that she did not develop this complication. Others have also reported the same favourable outcome in two sets of monochorionic twins in a quadruplet pregnancy [10]. 

In a study that consisted of 464 multiple gestations, monochorionic pairs were found more commonly in naturally conceived pregnancies than in those resulting from assisted reproductive technology. The frequency of monochorionic pairs after IVF with blastocyst transfer was double the frequency from IVF with cleavage stage transfer on day 3, but the difference was not statistically significant [19]. The embryos we transferred were at cleavage stage and were transferred on luteal day 3. 

Selective foetal reduction has been advocated to improve the outcome in high-order multiple pregnancies, with less foetal loss rate, prematurity, infant morbidity, and mortality [20, 21]. On the other hand, others found that high-order multiple pregnancies after foetal reduction is still associated with mild increased risk of premature delivery and low birth weight compared to nonreduced twin pregnancies [22]. Others also supported this observation when they found that the pregnancy duration and birth weight were inversely related to the initial gestational sac number irrespective of the final birth number in spontaneously reduced multifoetal gestations [23]. Our patient declined such intervention; not just because of the above debate, but also after she was informed about the high spontaneous foetal reduction rate that was reported in up to 40% to 65% of quadruplets [23, 24], and in up to 16.7% for the entire quadruplets pregnancies [13]. 

What is most important to know is that most of these complications (RDS, Mortality, others) are gestational age dependant. In a review of 112 multiple pregnancies to determine the maternal and neonatal outcome of multifetal pregnancies under a conservative pregnancy management, mortality and morbidity in triplets and higher-order multiples (quadruplets and quintuplets) were related to preterm delivery and did not exceed the rates of singletons or twins of an identical gestational age [12]. In a large study of multiple pregnancies that involved all Swiss hospitals, a comparison of triplets with matched singletons showed no significant differences in morbidity and mortality [1]. Our case was a good example of this, as she did not sustain any of these complications, and this could simply be due to the advanced gestational age she reached. Therefore, all efforts should be taken to extend the gestational age of higher order pregnancies, when possible. 

Although our patient was lucky in not developing significant maternal complication and she tolerated the physical and emotional burden of carrying a quadruplets to this advanced gestational age, which led to the good outcome her babies had; limiting the number of embryos transferred, especially to patients with good prognosis, should always be practiced to minimize any potential complications as a result of multiple gestation.
